# Activation of κ-opioid receptor by U50,488H improves vascular dysfunction in streptozotocin-induced diabetic rats

**DOI:** 10.1186/s12902-015-0004-7

**Published:** 2015-02-28

**Authors:** Xuan Zhou, Dongjuan Wang, Yuyang Zhang, Jinxia Zhang, Dingcheng Xiang, Haichang Wang

**Affiliations:** 1 Department of Cardiology, Xijing Hospital, Fourth Military Medical University, Xi’an, Shaanxi 710032 China; 2 Department of Cardiology, General Hospital of Guang Zhou Military Command, Guangzhou, Guangdong 510011 China; 3 Department of Cardiology, PLA Navy General Hospital, Beijing, 100048 China

**Keywords:** κ-opioid receptor, Vascular dysfunction, Inflammation, Diabetes mellitus

## Abstract

**Background:**

Evidence suggests that activation of κ-opioid receptor (KOR) by U50,488H exhibits potential cardiovascular protective properties. However, the effects of U50,488H on vascular dysfunction in diabetes mellitus (DM) are still not clear. The present study was designed to investigate the effects of U50,488H on vascular dysfunction in diabetic rats and explore the underlying mechanisms involved.

**Methods:**

Rats were randomly divided into control, DM, DM + vehicle, DM + U50,488H and DM + nor-binaltorphimine (nor-BNI) groups. Streptozotocin injection was used to induce DM. Weight, blood glucose, blood pressure and plasma insulin for each group were measured. Arterial functions were assessed with isolated vessels mounted for isometric tension recordings. Angiotensin II (ANG II), soluble intercellular adhesion molecule-1 (sICAM-1), interleukin (IL)-6 and IL-8 levels were measured by ELISA, and endothelial nitric oxide synthase (eNOS) phosphorylation and NF-κB p65 translocation were measured by Western blot.

**Results:**

Activation of KOR by U50,488H reduced the enhanced contractility of aortas to KCl and noradrenaline and increased acetylcholine-induced vascular relaxation, which could also protect the aortal ultrastructure in DM. U50,488H treatment resulted in reduction in ANG II, sICAM-1, IL-6 and IL-8 levels and elevation in NO levels, while these effects were abolished by nor-BNI treatment. Further more, eNOS phosphorylation was increased, and NF-κB p65 translocation was decreased after U50,488H treatment.

**Conclusions:**

Our study demonstrated that U50,488H may have therapeutic effects on diabetic vascular dysfunction by improving endothelial dysfunction and attenuating chronic inflammation, which may be dependent on phosphorylation of eNOS and downstream inhibition of NF-кB.

## Background

Diabetes mellitus (DM) has become an increasing burden on human health worldwide [1]. It is reported that cardiovascular complications are the leading etiology of morbidity and mortality in the diabetic population [2,3]. Growing evidence has indicated that vascular dysfunction is one of the most important causes for diabetic cardiovascular disease [4]. However, until now there are still few effective ways to treat diabetic vascular dysfunction.

Opioid receptors, discovered by Solomon H. Snyder in 1973, belong to the G protein-coupled receptor superfamily [5]. There are three principal subtypes of opioid receptors: μ-opioid receptor, δ-opioid receptor, and κ-opioid receptor (KOR) [6]. It is reported that opioid receptors are widely expressed throughout the body, including the brain, heart, digestive tract and vessels [7-9], and can be activated by the endogenous opioid peptide dynorphin [10].

It has been shown that KOR is the predominant type of opioid receptors in the cardiovascular system [11], and has cardioprotective and anti-arrhythmic effects during myocardial ischemia/reperfusion [12,13]. A study using hypoxic pulmonary hypertensive rats found that KOR activation was associated with improved vascular function [14]. Therefore, we hypothesized that KOR activation may have therapeutic potential for the treatment of diabetic vascular dysfunction.

Recent data have suggested that U50,488H is a highly selective KOR agonist with lower abuse potential and fewer side effects, such as respiratory depression, convulsions and gastrointestinal intolerance [11,15]. Upon activation, KOR could regulate a number of key signaling pathways [16-18], which are implicated in cardiovascular protection. Additionally, nor-binaltorphimine (nor-BNI) was found to be an antagonist that blocked KOR without affecting the μ-opioid receptor and the δ-opioid receptor [19].

It is well known that endothelial dysfunction and low-grade inflammation play important roles in vascular dysfunction [20-22]. Endothelium can release multiple vasoactive factors, including angiotensin II (ANG II), nitric oxide (NO), soluble intercellular adhesion molecule-1 (sICAM-1), interleukin (IL)-6 and IL-8, to maintain vascular homeostasis [23-25]. Elevated levels of ANG II are associated with vascular remodeling [26], while reduced NO levels could be involved in the development of diabetic angiopathy [27]. Vascular studies on DM have found low-grade inflammation in the vascular wall, which may result in increased vascular stiffness and blood flow retardation [28]. It has been recognized that NF-κB signaling plays an important role in inflammation in many diseases. Recent studies also reveal that NF-κB signaling may be involved in the KOR activation-offered protective effects [29,30].

In this study, we investigated whether KOR activation was involved in attenuating diabetic vascular dysfunction by U50,488H and explored the downstream molecular mechanisms.

## Methods

### Animals

In order to establish diabetic model, streptozotocin (STZ, Sigma, St. Louis, MO, USA) injection was applied. Male Sprague–Dawley (SD) rats (weight 200–240 g) received intraperitoneal injection of STZ (35 mg/kg) for 3 days [31]. Blood glucose levels were tested by blood glucose meter (Lifescan, Inc., Milpitas, CA, USA) one week following STZ injection. Animals with glucose levels ≥16.6 mmol/L were considered as diabetic. Diabetic rats received standard food for 4 weeks and then randomized into the following 4 groups (n = 15): (1) DM group: diabetic rats without any treatment; (2) DM + U50,488H group: diabetic rats that received daily treatment of U50,488H (Biomol, Plymouth Meeting, PA, USA) at 1 mg/kg for 4 weeks; (3) DM + vehicle group: diabetic rats that received only saline daily for 4 weeks; (4) DM + nor-BNI group: diabetic rats that received daily treatment of nor-BNI (Sigma, St. Louis, MO, USA) at 0.5 mg/kg for 4 weeks. Age-matched normal rats that received only saline daily (n = 15) were used as non-diabetic controls (CON). All experiments were conducted under the National Institutes of Health Guidelines on the Use of Laboratory Animal and were approved by the Fourth Military Medical University Committee on Animal Care.

### Measurement of blood pressure and insulin levels

Blood pressure was monitored with a tail cuff system (Non-invasive Blood Pressure System, PanLab) as described previously [32]. Briefly, rats were placed in a warm chamber (37°C) for 10 minutes to rest, and then occluding cuffs and pneumatic pulse transducers were placed on the tails. Five readings were obtained from each rat. Commercially available ELISA kit was used to determine plasma insulin levels (Cusabio Biotech Co., China).

### Detection of KOR expression in thoracic aortas

To detect the KOR expression in thoracic aorta, immunohistochemical staining was performed. Briefly, the thoracic aortas were isolated, fixed by 4% paraformaldehyde and paraffin embedded. Serial tissue sections (5 μm) were mounted on slides, then xylene and ethanol were used to deparaffinize and dehydrate the tissue respectively. Non-specific antigens were blocked by 20% normal bovine serum for 30 minutes, which was followed by treatment with 0.3% hydrogen peroxide. Then the sections were incubated overnight at 4°C with KOR antibody (1:100, Santa Cruz Biotechnology, Santa Cruz, CA, USA). After three washes in PBS, the sections were incubated with a biotinylated secondary antibody (1:2000, Santa Cruz Biotechnology, Santa Cruz, CA, USA) for 1 hour at 37°C and washed again in PBS. Then horseradish peroxidase was developed with 3,3-diaminobenzadine (DAB) (Roche diagnostics, Mannheim, Germany) as the chromogen substrate. After rinsing, the slides were mounted with cover slips, and the positive samples displayed a brown color under a light microscope.

### Transmission electron microscope analysis of thoracic aortas

Transmission electron microscope (TEM) was performed to identify the ultrastructural changes of thoracic aortas. After anesthetized, the thoracic aortas from the rats were immediately isolated and cut into 1–2 mm wide, and then kept in glutaraldehyde and osmium tetroxide in order. Subsequently, the TEM specimens were dehydrated using graded ethanol and embedded in resin. The ultrastructure of thoracic aortas were visualized by ultra-thin sections under transmission electron microscope (JEOL JEM-2000EX, Tokyo, Japan).

### Measurement of ANG II, sICAM-1, IL-6 and IL-8

Enzyme-linked immunosorbent assay (ELISA, Bionewtrans Pharmaciutical Biotechnology Co.Ltd, USA) was performed to determine the serum levels of ANG II, sICAM-1, IL-6 and IL-8, as previously described [33]. Standard curves were drawn by manufacturers’ instructions. Absorbance of standards and samples was measured by microplate reader (Model 550, Bio-Rad, Japan) at 450 nm and the serum levels of ANG II, sICAM-1, IL-6 and IL-8 were calculated according to the standard curves.

### Isometric tension recordings for thoracic aortas

The arterial functions were assessed with endothelium-intact isolated rats vessels mounted for isometric tension recordings in organ chambers, as described previously [34]. Rats were anesthetized with pentobarbital sodium (40 mg/kg, i.p.), followed by the rapid removal of thoracic aortas. The vessels were cleared of excess connective tissues under a stereomicroscope and cut into rings (diameter ≈ 3 mm).

Vascular rings were mounted on a myograph (Multi Wire Myograph System-610 M, Danish Myo Technology A/S, Denmark) under their optimal resting tension (1.0 g) in 15 ml organ baths containing warmed (37°C), oxygenated (95% O_2_ + 5% CO_2_) Krebs solution (118.3 mmol/L NaCl, 4.7 mmol/L KCl, 25 mmol/L NaHCO3, 1.2 mmol/L MgSO4, 1.2 mmol/L KH2PO4, 2.5 mmol/L CaCl2, 11.1 mmol/L glucose and 0.026 mmol/L EDTA, pH 7.4). The vessels were allowed to equilibrate for 60 minutes before the onset of experiments, with Krebs solution changed every 15 minutes. After a washout period, vascular rings were challenged with a cumulative concentration of KCl (10–90 mmol/L) or noradrenaline (NE, 10^−9^-10^−4^ mol/L) to test vasoconstrictive activity. In addition, arterial contractions were induced by phenylephrine (PE, 80 μmol/L), and then were relaxed by acetylcholine (ACh, 10^−9^-10^−4^ mol/L) or sodium nitroprusside (SNP, 10^−9^-10^−4^ mol/L) to test vasodilative activity. Myograph data were recorded by PowerLab and Chart software (AD Instruments, Australia), and cumulative concentration response curves were then generated.

### Western blot analysis for protein expression of KOR, eNOS, and NF-κB p65

Western blot was performed as previously described [31]. Briefly, the thoracic aortic tissues from all groups were quickly isolated and snap-frozen. Total proteins (for KOR and eNOS) were extracted with lysate and nuclear proteins (for NF-κB p65) were isolated using a nuclear NE-PER extract kit (Thermo Scientific, IL, USA). Equal weight of proteins (40 μg) were loaded into a SDS-PAGE gel. After separated, the proteins were transferred onto nitrocellulose membranes electrophoretically. Then the membranes were incubated with 5% skim milk for 1 hour, appropriate primary antibody at 4°C overnight, and secondary antibody at room temperature for 1 hour in order. Enhanced chemiluminescence reagent kit (Millipore) was used for development of the blots, which were detected with UVP Bio-Imaging Systems. Protein levels were evaluated by Vision Works LS Acquisition and Analysis Software.

The following primary antibodies were used: KOR (1:1 000), endothelial nitric oxide synthase (eNOS, 1:1 000), phospho-eNOS (at Ser-1177) (1:1 000), NF-κB p65 (1:1 000), histone 3 (1:1 000) and β-actin (1:1 000). Secondary antibodies were horseradish peroxidase-conjugated goat anti-rabbit IgG and rabbit anti-goat IgG at 1:5,000 dilution. All above antibodies were purchased from Santa Cruz Biotechnology.

### Statistical analysis

All statistical analysis were performed by GraphPad Prism software version 5.0 (GraphPad Software, CA, USA). Values were presented as mean ± SD and analyzed using ANOVA with Bonferroni corrected t-test. Western blot results were analyzed using the Kruskal–Wallis test followed by Dunn’s post hoc test. Differences were considered statistically significant if P < 0.05.

## Results

### Basic parameters of rats in different experimental groups

Administration of U50,488H or nor-BNI was initiated 4 weeks following the onset of experimental DM. The metabolic characteristics of rats in different groups are shown in Table 1. Diabetic rats exhibited hyperglycemia (22.3 ± 2.19 mmol/L *vs.* 8.2 ± 0.94 mmol/L, P < 0.05), decreased body weight gain (286.6 ± 14.61 g *vs.* 419.7 ± 12.25 g, P < 0.05) and reduced insulin levels (0.51 ± 0.093 ng/mL *vs.* 2.42 ± 0.466 ng/mL, P < 0.05) compared with CON group. There was no significant difference in blood pressure (103.6 ± 6.40 mmHg *vs.* 104.9 ± 6.14 mmHg, P > 0.05) between CON and DM groups. Neither U50,488H nor nor-BNI administration influenced body weight, blood glucose levels, blood pressure and insulin levels in diabetic rats.

**Table 1:** **Basic parameters of rats in different experimental groups**

Characteristics	CON	DM	DM + U50,488H	DM + vehicle	DM + nor-BNI
**Baseline**					
Weight (g)	222.7 ± 10.17	225.2 ± 8.24	224.6 ± 8.87	220.2 ± 7.12	221.5 ± 9.60
Blood pressure (mmHg)	102.8 ± 6.56	104.7 ± 6.86	100.5 ± 5.50	103.9 ± 5.38	103.1 ± 5.66
Blood glucose (mmol/L)	7.9 ± 0.96	21.5 ± 2.67*	23.66 ± 2.65*	22.1 ± 3.57*	21.64 ± 2.46*
Plasma insulin (ng/ml)	2.29 ± 0.446	0.53 ± 0.077*	0.63 ± 0.125*	0.50 ± 0.061*	0.59 ± 0.116*
**After 4 weeks of treatment**					
Weight (g)	419.7 ± 12.25	286.6 ± 14.61*	281.3 ± 17.44*	284.2 ± 12.45*	284.7 ± 16.66*
Blood pressure (mmHg)	103.6 ± 6.40	104.9 ± 6.14	102.3 ± 6.23	107.9 ± 5.41	104.5 ± 6.47
Blood glucose (mg/dL)	8.2 ± 0.94	22.3 ± 2.19*	21.4 ± 1.67*	22.8 ± 2.28*	22.7 ± 1.93*
Plasma insulin (ng/ml)	2.42 ± 0.466	0.51 ± 0.093*	0.58 ± 0.109*	0.50 ± 0.075*	0.56 ± 0.134*

**Legend:** Data are mean ± SD (n = 15), *P < 0.05 *vs.* CON group.

### KOR expression was increased in thoracic aortas of DM rats

**Figure 1:**
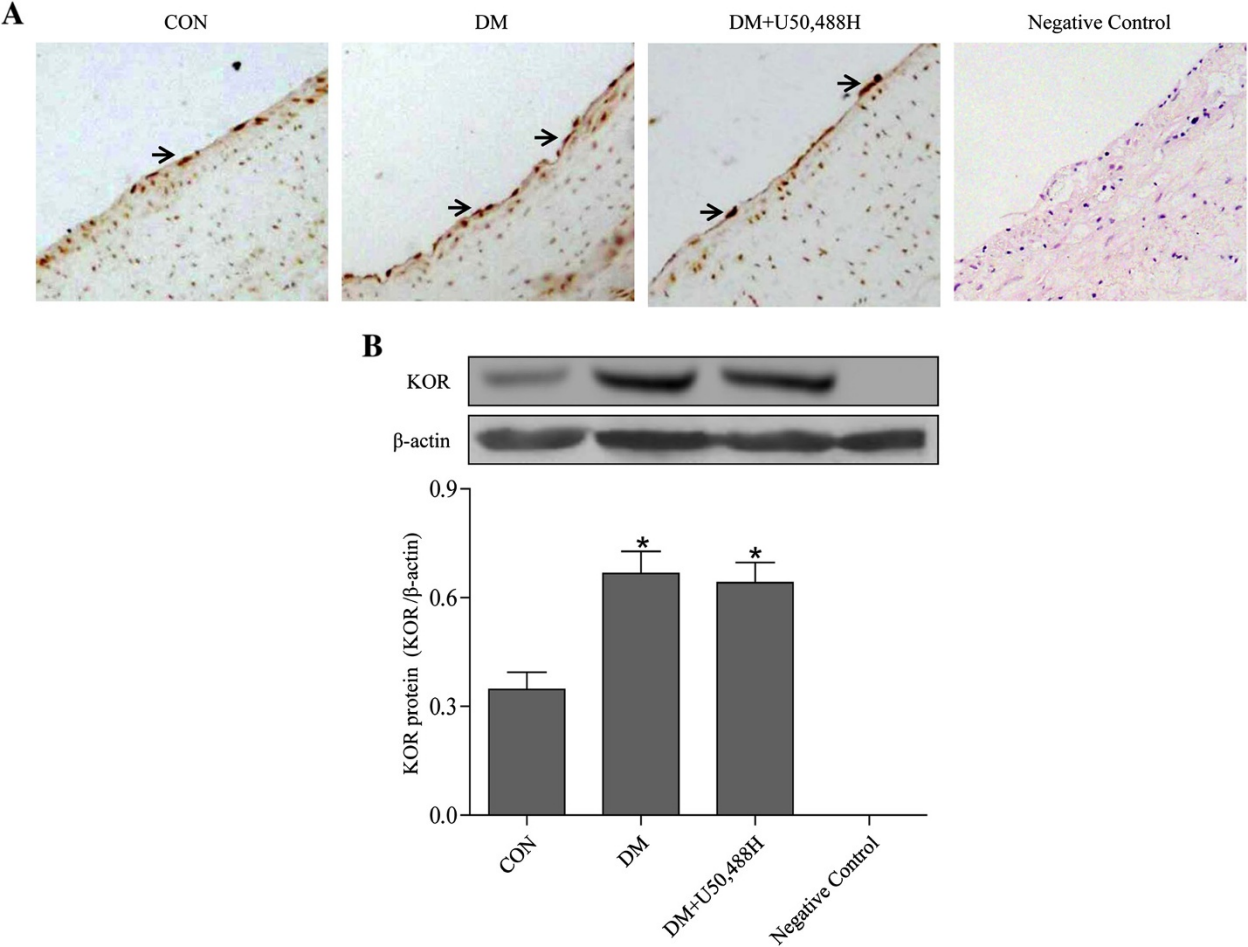
**KOR was expressed in thoracic aortas. (A)** Expression of KOR by immunohistochemical staining. KOR was mainly detectable in the endothelium and smooth muscle of thoracic aortas, highlighted with arrows. (scale bar: 25 μm). **(B)** Western blot analysis for KOR protein expression. *P < 0.05 *vs.* CON group.

As shown in Figure 1A, KOR was mainly detectable in the endothelium and smooth muscle of the thoracic aortas. Compared with the CON group, the KOR protein expression increased significantly in the DM group, which was also confirmed by Western blot (Figure 1B). We further tested KOR expression in the DM + U50,488H group and found no statistically significant difference compared with the DM group (Figure 1C).

### KOR activation improved vasoconstrictive function of thoracic aortas in DM rats

**Figure 2:**
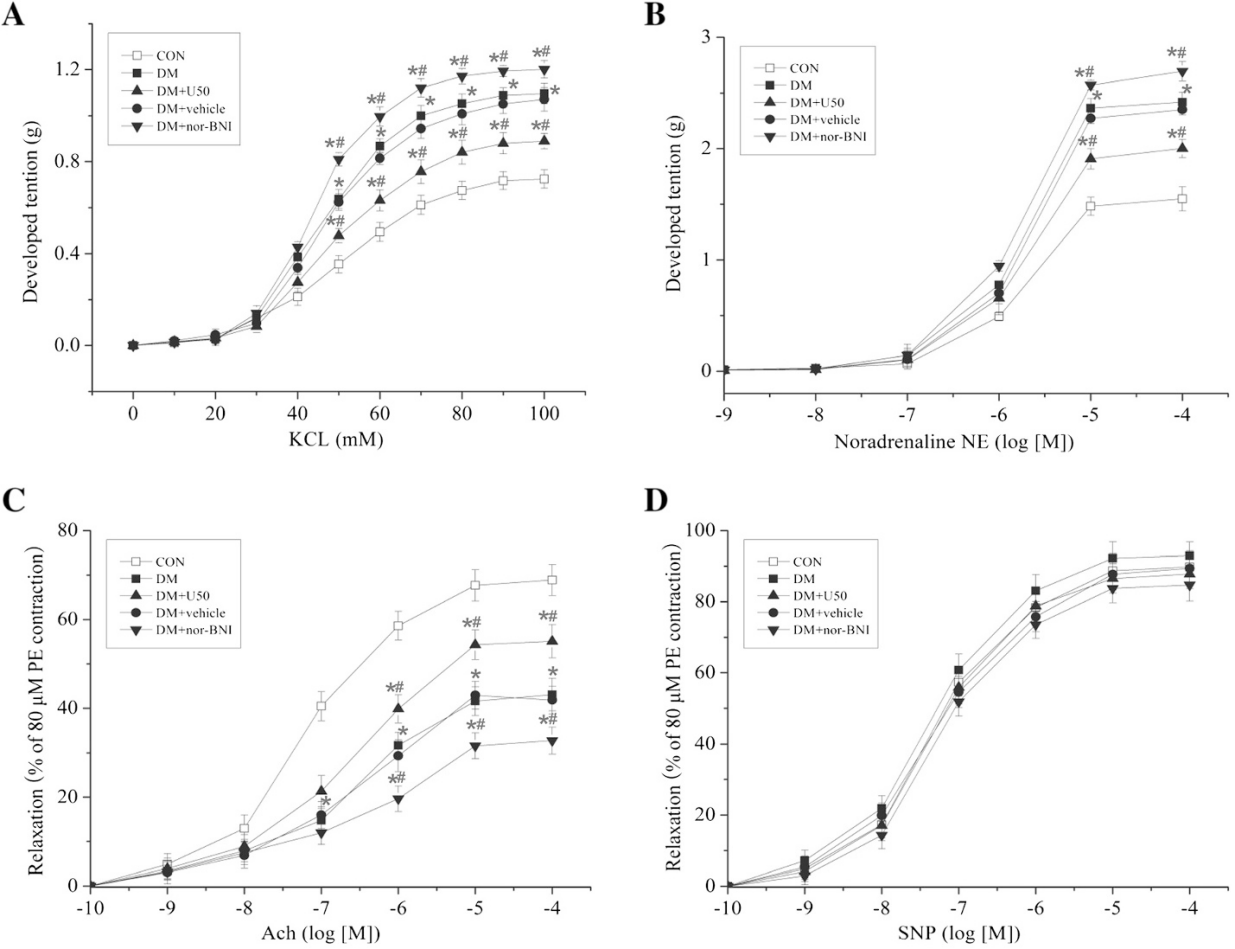
**U50,488H improved vasoconstrictive and vasodilative functions of thoracic aortas in DM rats.** Concentration-response curves for KCl (0–100 mmol/L) **(A)** or NE (10^−9^-10^−4^ mol/L) **(B)** induced vasoconstriction. U50,488H administration induced a rightward shift in the concentration-response curves, while nor-BNI administration induced a leftward shift in the concentration-response curves. **(C)** Concentration-response curves for ACh-induced vasodilation showed that U50,488H treatment improved vasodilative function of thoracic aortas. **(D)** Concentration-response curves for SNP-induced vasodilation showed no statistical differences among all five groups. *P < 0.05 *vs.* CON group, ^#^P < 0.05 *vs.* DM group. (n = 5).

To determine the effects of KOR activation on vasoconstrictive function of thoracic aortas in DM rats, isometric tension recordings were performed. The concentration-response curves for KCl (0–100 mmol/L) and NE (10^−9^-10^−4^ mol/L) are shown in Figure 2A-B. The vasoconstrictive function of thoracic aortas was significantly greater for the DM group compared with CON group. U50,488H administration induced a marked rightward shift in concentration-response curves, while nor-BNI administration induced a marked leftward shift in concentration-response curves, indicating that KOR activation was involved in attenuation of the vasoconstrictive abnormalities in DM rats. However, we also found that U50,488H administration could not restore the vasoconstrictive function to the normal level.

### KOR activation improved vasodilative function of thoracic aortas in DM rats

Vasodilative function was determined by isometric tension recordings with addition of ACh (10^−9^-10^−4^ mol/L) or SNP (10^−9^-10^−4^). Concentration-response curves (Figure 2C) for ACh revealed that DM led to lower vasodilatory potency, which was significantly improved with U50,488H treatment. In contrast, nor-BNI administration further aggravated the vasodilative abnormalities of diabetic rats. However, we also found that U50,488H administration could not restore the vasodilative function to the normal level. There were no statistically significant differences in relaxation induced by SNP administration among all five groups (Figure 2D). Taken together, all these data indicated that KOR activation by U50,488H could improve the vasodilative function in DM endothelium-dependently.

### KOR activation protected thoracic aortal ultrastructure in DM rats

**Figure 3:**
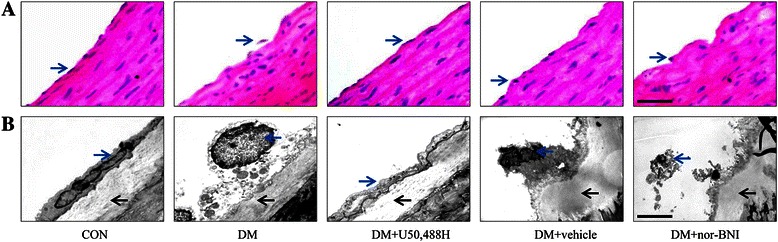
**U50,488H protected thoracic aortal structure in DM rats. (A)** HE staining of the aorta. The internal surfaces of thoracic aortas were smooth and well-integrated. The endothelium was falling in the DM group, while less endothelium was falling in the DM + U50,488H group. (scale bar: 25 μm). **(B)** Ultrastructure of the thoracic aortas under TEM (scale bar: 2 μm). In the DM group, the endothelium of the aorta was detached from the smooth muscle, the endothelial cell junctions were not integrated, smooth muscle cells migrated into the endothelium, and more collagen fibrils and irregular thickening of elastic fibers were observed. U50,488H treatment attenuated the changes. Endothelial cells are indicated with blue arrows, and internal elastic lamina is indicated with black arrows.

Hematoxylin and eosin (HE) staining showed intact endothelium in the CON group, that the endothelium was falling in the DM group, and only part of the endothelium was falling in the DM + U50,488H group (Figure 3A). Transmission electron microscope analysis was performed to further evaluate the effects of KOR activation on the thoracic aortal ultrastructure in DM rats. In the CON group, the surface of the endothelium was smooth, well-integrated and tightly affixed to the underlying smooth muscle. However, the endothelium of aortas from the DM group showed signs of degeneration, such as swelling and necrosis, and was noticeably detached from the smooth muscle. The endothelial cell junctions were not integrated, and smooth muscle cells migrated into the endothelium. Additionally, more collagen fibrils and irregular thickening of elastic fibers were observed in the pericellular spaces of enlarged smooth muscle cells. U50,488H treatment effectively attenuated the changes, preserving the integrity of the endothelium and decreasing smooth muscle cells migration, while treatment with vehicle or nor-BNI did not show these positive effects (Figure 3B).

### KOR activation reduced serum levels of NO and ANG II in DM rats

**Figure 4:**
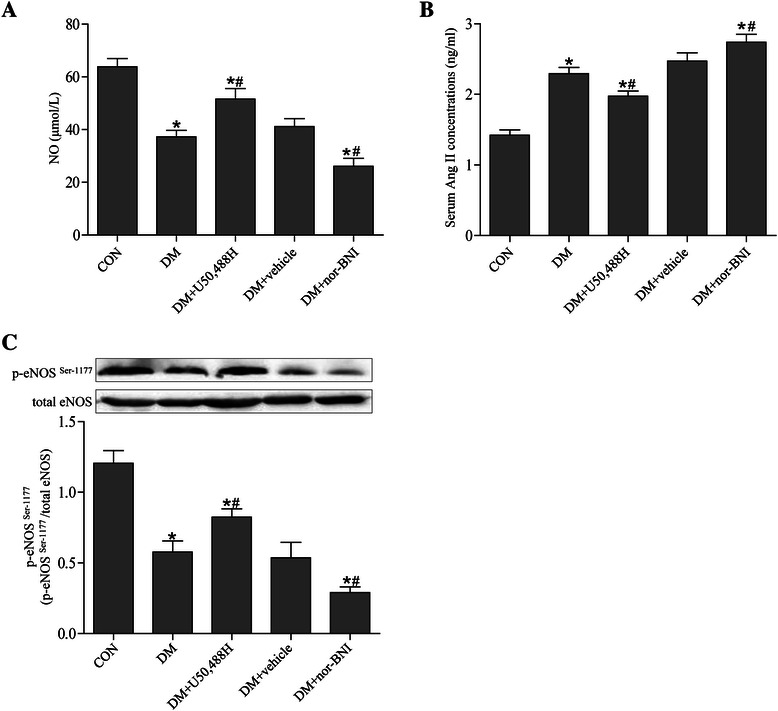
**U50,488H increased serum NO levels and reduced ANG II levels in DM rats. (A)** ELISA analysis showed significant decreases in serum NO levels in DM rats as compared with CON group. Treatment with U50,488H increased NO levels in DM. **(B)** ELISA analysis showed significant increases in serum ANG II levels in DM rats as compared with CON group, and U50,488H treatment blocked this elevation. **(C)** eNOS phosphorylation in DM rats was decreased in DM group and markedly increased after U50,488H treatment. *P < 0.05 *vs.* CON group, ^#^P < 0.05 *vs.* DM group. (n = 5).

Serum NO concentrations in the DM group were significantly decreased compared with the CON group (Figure 4A). Treatment with U50,488H enhanced NO secretion, while treatment with vehicle did not have this positive effect. Additionally, nor-BNI treatment resulted in less NO secretion. ANG II levels in the DM group were higher compared with the CON group, while U50,488H treatment attenuated ANG II production, and nor-BNI treatment further aggravated ANG II production (Figure 4B). We also investigated the effect of U50,488H on eNOS phosphorylation in thoracic aortas. The DM group showed decreased eNOS phosphorylation, while U50,488H treatment increased eNOS phosphorylation (Figure 4C). Furthermore, in the DM + U50,488H group, NO levels and eNOS phosphorylation were lower and ANG II levels were higher compared with the CON group.

### KOR activation suppressed DM-induced inflammatory response through NF-кB inhibition

**Figure 5:**
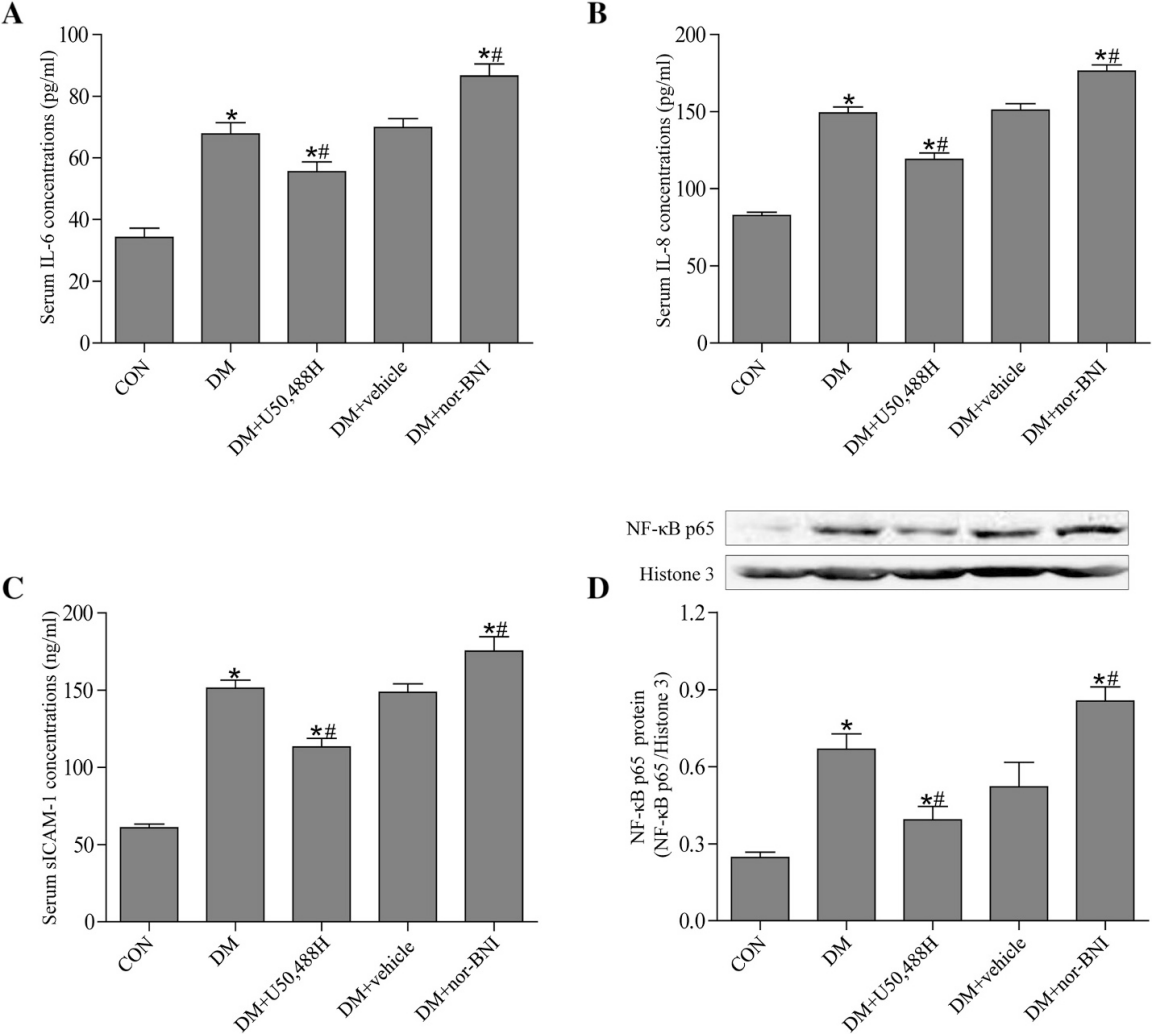
**U50,488H suppressed DM-induced inflammatory response through NF-кB inhibition.** ELISA analysis showed significant increases in serum levels of IL-6 **(A)**, IL-8 **(B)**, and sICAM-1 **(C)** in DM rats as compared with the CON group, and U50,488H treatment prevented these increases. **(D)** NF-κB p65 translocation was increased in DM rats as compared with the CON group, which was markedly attenuated by U50,488H treatment. *P < 0.05 *vs.* CON group, ^#^P < 0.05 *vs.* DM group. (n = 5).

To determine whether U50,488H inhibited the DM-induced inflammatory response in rats, pro-inflammatory cytokines and adhesion molecule levels were measured. As shown in Figure 5A-C, concentrations of IL-6, IL-8 and sICAM-1 increased markedly in the DM group compared with the CON group. Treatment with U50,488H inhibited DM-induced IL-6, IL-8 and sICAM-1 production but did not restore them to the normal levels, while nor-BNI treatment further increased IL-6, IL-8 and sICAM-1 production.

It is reported that inhibition of the transcription factor NF-кB is important in mediating anti-inflammatory activity. Therefore, Western blot was performed to investigate NF-кB nuclear translocation. The results showed that nuclear translocation of p65 protein induced by DM was markedly attenuated by U50,488H treatment, while nor-BNI treatment increased nuclear translocation of p65 protein (Figure 5D).

## Discussion

It is reported patients with DM show two- to four-fold more cardiovascular events compared with patients without DM [35]. It has been proposed that the reason for the increased incidence may be closely related to vascular dysfunction. Vascular dysfunction in DM is evident from impaired vasodilative response, endothelial cells dysfunction, dysregulated cytokine secretion, and hemodynamic disorder [36]. An effective way to treat diabetic vascular dysfunction is critical. In this study, we investigated whether activation of KOR by U50,488H could improve vascular dysfunction in diabetic rats.

There are two main types of diabetes: insulin-dependent DM (type 1) and non-insulin-dependent DM (type 2). Type 1 DM is strongly associated with autoimmunity, while type 2 DM is strongly associated with obesity and insulin resistance. STZ-induced DM offers a very cost effective and expeditious technique for DM research. STZ also offers the additional benefit of being able to select specific traits of interest, which can be important for specific experimental design [37]. In this study, the DM model was induced by intraperitoneal injection of STZ (35 mg/kg) for 3 days, which is useful to examine the beneficial effects of U50,488H against high glucose-induced vascular dysfunction *in vivo*, and could be devoid of potential confounding effects of obesity (type 2 DM) or autoimmunity (type 1 DM) [38,39].

Previous research has indicated that opioid receptors are widely distributed in the cardiovascular system [40]. Opioid receptors, especially KOR, have many cardiovascular protective effects, such as reducing myocardial ischemia/reperfusion injury, anti-arrhythmia function and attenuating hypoxic pulmonary artery hypertension [12,41,42]. Results from the current study demonstrated that KOR was mainly expressed in the endothelium and smooth muscle of the thoracic aortas, and that the KOR expression was increased significantly in the DM group compared with the CON group. These data suggest that KOR activation may play a role in protecting against aortic dysfunction induced by DM. At the mean time, KOR activation by U50,488H did not influence the blood glucose levels in DM rats, indicating that the protective effects of U50,488H were independent of blood glucose lowering.

We further found that KOR activation could improve vasoconstrictive function in DM, evidenced by a reduction in vascular contractility to NE and KCl. We also observed that the impaired endothelium-dependent vasorelaxation in response to ACh in DM was ameliorated significantly by KOR activation, which suggests that the attenuation of endothelial dysfunction by KOR activation may, at least in part, account for the improvement in vasorelaxation.

Endothelial dysfunction has been recognized as one of the major causes involved in the development of cardiovascular diseases [43]. Clinical studies have demonstrated that endothelial impairment in DM is the first step in vascular dysfunction [44]. Our results further conform that KOR activation by U50,488H can effectively preserve the integrity of the endothelium in thoracic aortas of DM rats.

Endothelium can maintain vascular homeostasis through multiple regulatory pathways that involve the release of vasoactive factors such as NO and ANG II [45,46]. NO is a critical biological messenger and effector molecule, which is involved in mediating many physiological functions in the cardiovascular system [47]. We found that treatment with U50,488H enhanced NO secretion, while treatment with nor-BNI resulted in less NO secretion. U50,488H treatment could restore the balance between the vasodilative and vasoconstrictive factors in endothelium, as the response of thoracic aortas to SNP showed no statistically significant difference.

We further found that KOR activation by U50,488H reduced serum levels of ANG II. As we known, ANG II exerts an important action in mediating endothelial dysfunction, vascular inflammation, hypertrophy and remodeling. Park *et al.* reported that ANG II contributed to impairment of insulin-stimulated vasodilation in vascular endothelium in DM rats [48]. Down-regulation of ANG II played a critical role in inhibition of vascular remodeling and endothelial dysfunction in DM [26,49].

To explore the mechanism by which U50,488H improved DM-induced endothelial dysfunction, we further investigated eNOS phosphorylation. Evidence showed that eNOS phosphorylation was associated with the increase in NO production, as well as the decrease in ANG II production [50]. It has been proved that KOR agonists could significantly increase the level of NO and decrease the level of ANG II in blood plasma of hypertensive rats [51]. Consistent with previous studies, we found that DM caused significant reductions in eNOS phosphorylation, while U50,488H treatment increased eNOS phosphorylation. These findings suggest that U50,488H improved endothelial dysfunction via a mechanism that involves an action of increasing eNOS phosphorylation by activating KOR.

Chronic inflammation is another important cause accounting for vascular dysfunction. Accumulating evidence suggests that U50,488H has therapeutic potential in cardiovascular protection through an anti-inflammatory response, which exhibits a broad inhibitory influence on cytokines, chemokines and chemokine receptor expression. Wu *et al.* reported that U50,488H administration could inhibit neutrophil accumulation and TNF-α induction in myocardium subjected to ischemia/reperfusion [52]. Lin *et al.* reported that U50,488H administration could inhibit TLR4/NF-κB signaling induced by ischemia/reperfusion in rats hearts [30]. In this study, we found that treatment with U50,488H inhibited DM-induced IL-6, IL-8 and sICAM-1 production. It is reported that IL-6, IL-8 and sICAM-1 are closely related to endothelial dysfunction, which subsequently results in the progression of vascular dysfunction [53].

We further investigated the mechanisms responsible for U50,488H-associated vascular protection in DM. It is reported that NF-κB plays a central role in the development of inflammation through regulation of genes encoding not only pro-inflammatory cytokines, but also adhesion molecules [34]. Our results showed that nuclear translocation of p65 protein induced by DM was markedly attenuated by U50,488H treatment, while nor-BNI treatment increased nuclear translocation of p65 protein. From these data, it is suggested that KOR activation could attenuate chronic inflammation in diabetic rats through NF-κB inhibition.

This study provides evidence supporting the protective effects of KOR activation by U50,488H against DM-induced vascular dysfunction. These findings indicate that KOR activation may represent a promising therapeutic strategy to maintain vascular function in patients with DM. Nonetheless, it is noteworthy that our findings were mainly based on rodent models and *in vitro* experiments. Therefore, further study is needed to evaluate the effects of KOR activation on vascular function in patients with DM.

## Conclusion

KOR activation may have therapeutic effects on diabetic vascular dysfunction by attenuating chronic inflammation and improving endothelial function, which may be dependent on eNOS phosphorylation and downstream inhibition of NF-кB.
